# Metallic Implant Surface Activation through Electrospinning Coating of Nanocomposite Fiber for Bone Regeneration

**DOI:** 10.1155/2023/1332814

**Published:** 2023-03-03

**Authors:** Amjed Al-Khateeb, Emad S. Al-Hassani, Akram R. Jabur

**Affiliations:** Materials Engineering Department, University of Technology, Baghdad, Iraq

## Abstract

There is a critical need in orthopedic and orthodontic clinics for enhanced implant-bone interface contact to facilitate the quick establishment of a strong and durable connection. Surface modification by bioactive multifunctional materials is a possible way to overcome the poor osteoconductivity and the potential infection of Ti-based implants. Ti-25Zr biometallic alloy was prepared by powder metallurgy technique and then coated by Nano-composite fiber using electrospinning. Ceramic Nanocompound (CaTiO_3_, BaTiO_3_) was used as filler material and individually added to polymeric matrices constructed from the blend of polycaprolactone/chitosan. Using optical microscopy, scanning electron microscopy (SEM), energy-dispersive X-ray spectroscopy (EDX), Fourier transform infrared spectroscopy (FTIR), and wettability, respectively, the morphology, chemical analysis, surface roughness, and contact angle measurements of the samples were evaluated. The result shows a significant improvement in cell viability, proliferation, and ALP activity for coated samples compared to noncoated samples. PCL/Chitosan/Nano-CaTiO_3_ (CA1) recorded remarkable enhancement from the surface-coated samples, demonstrating a significantly higher cell viability value after seven days of MC3T3-E1 cell culture, reaching 271.56 ± 13.15%, and better cell differentiation with ALP activity reaching 5.61 ± 0.35 fold change for the same culture time. PCL/Chitosan/Nano-BaTiO_3_ (BA1) also shows significant improvement in cell viability by 181.63 ± 17.87% and has ALP activity of 3.97 ± 0.67 fold change. For coated samples, cell proliferation likewise exhibits a considerable temporal increase; the improvement reaches 237.53% for (CA1) and 125.16% for (BA1) in comparison with uncoated samples (bare Ti-25Zr). The coated samples resist bacteria in the antibacterial test compared to the noncoated samples with no inhibition zone. This behavior suggests that a Nanocomposite fiber coat containing an active ceramic Nanocompound (CaTiO_3_, BaTiO_3_) promotes cell growth and holds promise for orthodontic and orthopedic bioapplication.

## 1. Introduction

Bone is a living tissue that is formed of a hierarchically structured composite of highly organized collagen bundles reinforced by hydroxyapatite nanocrystals [1]. A bone defect is a lack of bone tissue in the body where it should normally be. This could be caused by several congenital or acquired conditions, such as trauma, tumor resection, or infections [2]. Unlike other tissues, most bone injuries can heal spontaneously and without additional treatment due to the bone's ability to regenerate itself [3].

However, for most surgeons, treating critical bone defects with a length greater than 1.5 times the diameter of the long bone is challenging. Failure to cure a bone defect is primarily caused by bone loss and harm to the physiological environment [4]. Although autologous bone graft and bone allograft are the two most common options for bone regeneration, their clinical benefits are not guaranteed. This is because complications and morbidity are frequently encountered in patients. Therefore, research on alternative bone substitutes is still necessary, and synthetic bone substitutes are still considered for bone tissue regeneration. Synthetic bone substitutes are used because of their biocompatibility, osteoconductivity, and potential to overcome the previously mentioned limitations of autologous and allogenic bone grafts [2].

Materials used for bone regeneration should have the characteristics of favorable osteoinduction, good osteoconduction, regulated bioactivity, and an adequate degradation rate to imitate the native bone structure [5].

Because of their exceptional mechanical qualities, superior biocompatibility, and excellent corrosion resistance, commercially pure titanium and Ti-based alloys have been widely employed in hard tissue replacement metallic implants, especially in the orthopedic and dental professions [5–8]. Among the most recommended reasons for bone-implant failure are stress shielding, inadequate osseointegration, and a high potential for bacterial infections. Since the modulus of elasticity of the implant is significantly greater than that of the surrounding bone, stress shielding is typically caused by this difference. This substantial variance in the modulus of elasticity value prevents the necessary stress from being transferred to adjacent bone, which leads to bone resorption around the implant and, eventually, loosening and failure. Ti-based alloys with nontoxic alloying elements and a low modulus of elasticity have been widely produced to address the issue of stress shielding. The Ti-Zr alloy is one of the most commonly investigated titanium alloys with, a low elastic modulus and enhanced mechanical and biological properties. Porous titanium alloys have undergone significant development in recent years. Additionally, titanium alloys are produced in regulated porous structures, which successfully lower the implant's modulus of elasticity to levels comparable to those of natural bone. Also, it permits new bone to grow within that porous area and achieve mechanical interlocking [9, 10].

However, the increase of zirconium element in the Ti-Zr alloy enhances its mechanical properties, but Zr contents exceeding 25 wt.% prevent the formation of calcium phosphate, which is the main component of human bones [11, 12]. Also, caution must be taken when considering high Zr concentrations due to an increased susceptibility to pitting corrosion [13].

Ti-base alloys are bioinert materials, and if the implant has not been adequately integrated within the bone, fibrous tissue can readily form between them, reducing the long-term survival of the alloy in the body [14, 15]. Furthermore, postoperative implant infections remain a significant problem. They are produced by wound contamination after surgery and bacterial strains introduced into the surfaces of metallic implants and surgical instruments due to insufficient sanitation. Infections caused by medical devices increase healthcare expenses, cause patient misery, and, in some difficult situations, result in death [6, 16].

Bioactive composite coatings on the surface of implants with multiple functionalities to encourage bone formation while preventing bacterial infection play an important role in strengthening osseointegration, shortening healing time, and extending implant life [17, 18].

For this purpose, polymeric matrices filled with bioactive ceramic Nanoparticles are often used as composite biomaterials. With this strategy, the processability, biodegradability, and mechanical characteristics of polymers, which are already good for bone tissue, are made even better by adding a bioactive ceramic phase to mimic the composition of natural bone [3, 19].

Polycaprolactone was chosen as the polymeric matrix material due to its thermoelastic nature, low melting point, ease of processing, outstanding mechanical strength, and biocompatibility, as well as the fact that it is an FDA-approved biodegradable polymer. To improve stress crack resistance, hydrophilicity, degradation rate, and cell adherence, PCL can be combined with other polymers. PCL combined with other polymers, such as cellulose propionate and cellulose acetate butyrate, has been proven to alter the drug release rate from microcapsules [20–22].

Chitosan (CS) is a polysaccharide derived from chitin deacetylation. CS is a natural polymer with antibacterial properties, a high absorption capacity, biodegradability, and biocompatibility. When CS comes into contact with the living tissue, it interacts with several cellular processes during wound healing and has the potential to speed up the healing process [23, 24]. Thus, combining PCL and chitosan for the coating process is a desirable method for enhancing biological and mechanical performance compared to using the components individually [25, 26].

Because of its biocompatibility, increased apatite bonding, and stimulation of cell adhesion and proliferation, perovskite calcium titanate has lately been employed as the primary coating component on titanium implants. CaTiO_3_ has shown promising applications for bone regeneration because it creates phosphate ions and opposite surface charges in a simulated bodily fluid (SBF), which impact future bone-like apatite formation [5, 27].

Barium titanate, BaTiO_3_ (BTO), is a smart material with a piezoelectric property that generates electrical polarization in reaction to minute structural deformations. BTO is said to have biological properties, including strong biocompatibility when connected with living cells. As a result, it has been identified as a promising material for biomedical applications [28, 29].

In the present work, low-modulus metallic biomaterial Ti-25Zr was created using the powder metallurgy technique. The inert surface of the Ti-25Zr was activated with a novel multifunctional Nanocomposite fiber. The electrospinning method was used for fabricating hybrid inorganic and organic Nano-fibrous mats with a good bond to the substrate. Different Nano-particle ceramic (CaTiO_3_, BaTiO_3_), each with attractive characteristics for biomedical application, was added as filer material to the blend of PCL/Chitosan to prepare two solutions (PCL/Chitosan/Nano-CaTiO_3_, PCL/Chitosan/Nano-BaTiO_3_) for electrospinning coating the surface of Ti-25Zr. The coated and uncoated samples' surface morphology and elemental composition were studied. The cytocompatibility of composite coated Ti-25Zr and the noncoated samples was also evaluated by in vitro cell culture.

## 2. Materials and Methods

### 2.1. Materials

Titanium powder (sigma Aldrich 150–200 *μ*m 99.7 purity, USA), zirconium powder (sigma Aldrich 100–150 *μ*m 99.5 purity, USA), and chitosan Nanopowder (medium molecular weight, 90% deacetylated, APS 80 nm) were obtained from (Hongwu International Group, China). Polycaprolactone (M*η* = 80.000 sigma Aldrich USA), Nano-ceramic (CaTiO_3_, BaTiO_3_) all in range (80–100 nm) supply from (Jinan Boss Chemical Industry, China), Acids (acetic, formic, sulfuric, hydrochloric) and acetone were purchased from (Thomas Baker Chemicals, India) and were all analytical grade.

### 2.2. Substrate Material Preparation

A Ti–25Zr disc (15 mm in diameter, 2 mm in height) as the base metal was prepared using the powder metallurgy technique. Titanium powder was mixed with 25 wt.% zirconium powder for 6 hours, then the mixed powder was compressed at 500 MPa. The green compact disc was then sintered in a vacuumed furnace10^−3^torr at a range of 10 °C/min, held for 2 hours at 1300°C temperature, and left to cool in the furnace.

### 2.3. Surface Treatment of the Base Sample

The Ti-25Zr samples were mechanically polished with 120–1000 grit silicon carbide paper. The samples were cleaned for 20 minutes using an ultrasonic cleaning path with acetone followed by deionized water. The surface of the metal implant was made rougher and more energetic by treating it with a combination of H_2_SO_4_ : HCl : H_2_O(1 : 1 : 1) at 60°C for one hour. In addition, samples were alkaline-treated with 10 M NaOH at 60°C for 24 hours and left to dry overnight.

### 2.4. Porosity Test

The porosity of the sintered Ti–25Zr alloys is determined by an equation

(1) $$\begin{array}{c} p=1-\frac{\rho }{\rho_{0}}\times 100\%, \end{array}$$

where *ρ* is the apparent density of the alloy, which is determined by the liquid displacement method using Archimedes' principle. *ρ*_0_ is the nominal theoretical density of the corresponding alloy, calculated as follows:

(2) $$\begin{array}{c} \rho_{0}=\frac{1}{\left(A\%/\rho_{A}\right)+\left(B\%/\rho_{B}\right)}, \end{array}$$

where *A*% and *B*% are the mass fractions of elements *A* and *B*, and *ρ*_*A*_ and *ρ*_*B*_ are the theoretical density of *A* and *B*.

### 2.5. Mechanical Evaluation of the Base Sample

A Vickers digital microhardness tester (HVS-1000, Laryee Technology, China) with a load of 9.8 N and a dwell period of 15 s was used to measure the hardness of the samples. The local values from 10 points were used to find the average microhardness values. The compressive stress measurement was done at room temperature by the Brazilian method using (a universal tensile machine made in China by Instron) for a sample having (a 15 mm diameter and an 18 mm height). Lastly, the elastic modulus was found by using (an ultrasonic tester of type CCT-4 UK) and solving the following equations:

(3) $$\begin{array}{c} ʋ=1-\frac{1}{2}\frac{1}{1-{\left(C_{trans}/C_{long}\right)}^{2}},\\ E=2\rho \left(1+ʋ\right)C_{trans}^{2}, \end{array}$$

where *C*_trans_: is the wave speed transversely, *C*_long_: is the wave speed longitudinally, *ʋ*: is the Poisson modulus,:is modules of elasticity, *ρ* density of the material (the density value assumes an isotropic, homogenous, and nondispersive material. The error was estimated using error propagation with a 95% confidence level).

### 2.6. Chemical and Microstructural Characterization of the Base Sample

The crystal phase was characterized by X-ray diffraction performed with Cu Ka radiation operated at 40 kV and 40 mA at room temperature (XRD, 6000 Shimadzu, Japan). The microstructures and surface topography of the samples were examined by scanning electron microscopy (SEM, TESCAN VEGA3, Czech Republic). The chemical composition and homogeneity of sintered samples were examined using energy-dispersive spectrometry (EDX), efficiently combining SEM imaging with elemental composition analysis.

### 2.7. Preparation of Electrospinning Solutions

Chitosan 2% (w/v) was dissolved in 4/6 acetic/formic (v/v) acid (100 ml) using a hot plate magnetic stirrer for 12 hours at 50°C to make a Chitosan solution. PCL 8% (w/v) was added to the Chitosan solution and stirred for 3 hours until a clear and homogenous solution of the PCL/Chitosan blended forms. The Nano-CaTiO_3_ and Nano-BaTiO_3_ were added individually, each with 1% (w/v), and stirred for 1 hour to form two solutions. Each solution was then homogenized for 3 min using a homogenizer (Model 300VT Ultrasonic Homogenizer USA) to make the PCL/Chitosan/Nano-CaTiO_3_ (CA1) and PCL/Chitosan/Nano-BaTiO_3_ (BA1) solutions ready for electrospinning coating.

### 2.8. Electrospinning

The solutions were prepared and placed in a 5 ml syringe fitted with a blunt-end 22 G needle. Using an infusion pump, the fluid was expelled at a rate of 1 ml/h (KD Scientific Syringe Pump 200, USA). The needle tips' distance from the grounded sample was maintained at 10 cm. The needle was subjected to a high voltage of 20 kV. The relative humidity in the room ranged between 35–55%. Before the examination, the fibers were dried overnight and kept in a desiccator.

### 2.9. Characterization of Coatings

The microstructures of the electrospun fibers were sputtered with gold before being examined using a field emission scanning electron microscope (FESEM) (Inspect F-50, Spain) at an accelerating voltage of 15 kV utilizing secondary electrons (SE). At random points on each fiber, the diameters of the resultant fibers were measured. The existence of Nano (CaTiO_3_, BaTiO_3_) in the PCL/Chitosan polymer blend was confirmed using a dispersive energy X-ray (EDX).

### 2.10. ATR-FTIR

Fourier transform infrared spectroscopy ATR-FTIR (Bruker Tensor 27 IR, Germany) was used to investigate the functional chemical groups. The FTIR spectra of pure chitosan, pristine polycaprolactone, and different types of composite coating were recorded between (4000–500) cm^−1^ regions using a universal ATR sampling accessory.

### 2.11. Wettability Evaluation

The wettability of the coated/uncoated samples was tested using the sessile drop technique with DD water (Optical Contact Angle SL200KS, China). This procedure included dropping 1 *μ*ml of distilled water onto the coated surface and measuring the contact angle of the water for 10 seconds. The test for wettability was performed in triplicate, and the contact angle was measured using a camera-based contact angle meter.

### 2.12. Antibacterial Test

The inhibition zone technique was used to evaluate the antibacterial activity of electrospun (PCL/Chitosan/Nano-CaTiO_3_ and PCL/Chitosan/Nano-BaTiO_3_) fibers and bare Ti-25Zr sample. The bacterial species *Staphylococcus aureus* (*S. aureus*) and *Streptococcus mutans* (*Sterp. mutans*) were used as model microorganisms. Plates of nutrient agar were infected with 1 mL of a bacterial solution containing about 10^8^ CFU/mL using the spread plate technique. The Nano-fiber coating substrate was applied to the inoculation plates in the shape of a 1.5 cm-diameter circular layer and then incubated at 37°C for 24 hours. Inhibition zones were calculated by measuring the clear region around each electrospun Nanofiber sample.

### 2.13. In Vitro Cellmaterial Interactions

#### 2.13.1. Samples Sterilization

Before cell seeding, samples were UV-sterilized for 20 minutes, submerged in 75% ethanol for 1 hour, and rinsed with PBS at least three times, each for 15 minutes.

#### 2.13.2. Cell Culture

The MC3T3-E1 cell line was obtained from the Pasteur Institute (Tehran, Iran). Cells were cultured in a humidified incubator with 5% CO_2_ in the air at 37°C and maintained in Dulbecco's Modified Eagle Medium (DMEM; Gibco, Life Technologies, Waltham, MA, USA) supplemented with 10% fetal bovine serum (FBS; BioWest SAS, Nuaille, France) and 1% PSF (antibiotic antimycotic solution, Sigma-Aldrich®, St. Louis, MO, USA). When cells were 75% confluent, they were detached at 37°C in phosphate-buffered saline (PBS) containing 0.1% ethylenediaminetetraacetic acid (Merck, Darmstadt, Germany) and 0.25% trypsin (Gibco, Invitrogen, Waltham, MA, USA). After that, we resuspended the cells in DMEM supplemented with 10% FBS and 1% PSF.

#### 2.13.3. Cell Seeding

At a density of 10,000 cells per well on 24-well culture plates, five 40 *μ*l drops of the cell suspension were carefully distributed throughout the surface of the samples. Following a 30-minute attachment period, the medium was introduced to the cell/sample complexes.

#### 2.13.4. Cell Viability Assay

MC3T3-E1 cell viability on the samples was estimated using the AlamarBlue assay (Sigma-Aldrich, St. Louis, MO, USA); briefly, 1 ml of 10%(v/v) AlamarBlue solution was added to each well and incubated for about 4 hours, then the absorbance was determined at 530/560 nm using an ELISA Reader (Stat Fax-2100, Miami, FL, USA). The cell viability was quantified by dividing the sample's absorbance by the absorbance of the control on days 1, 3, and 7. Data were obtained from three independent experiments (*n* = 3).

#### 2.13.5. Cell Proliferation

MC3T3-E1 preosteoblast proliferation was evaluated by determining the cell number in the samples at days 1, 3, and 7 and using the AlamarBlue® fluorescent assay. At each time point, samples were transferred to a new plate, AlamarBlue® was added, and the fluorescence was measured. After performing the AlamarBlue® assay each day, samples were washed twice with PBS and incubated in the osteogenic medium in a humidified incubator with 5% CO_2_ at 37°C. Data were obtained from samples from three independent experiments (*n* = 3).

#### 2.13.6. Alkaline Phosphate Enzymatic Activity

Alkaline phosphatase (ALP) assay is an essential method of assessing osteogenesis differentiations. Alkaline phosphatase (ALP) activity was measured to determine the osteoblastic phenotype of MC3T3-E1 preosteoblasts on coated and noncoated samples. On days 3 and 7 of cell culture on the sample's surface, cells were lysed with milli-Q water and freeze-thawed three times to determine ALP activity, and protein content-nitrophenyl-phosphate (Merck, Darmstadt, Germany) at pH 10.3 was used as the substrate for ALP, as described earlier [30]. The plate was immediately read at 405 nm using a spectrophotometer (BioTek, Winooski, VT) to obtain an absorbance reading correlated with the expression of (pNPP). ALP activity was calculated by dividing the amount of paranitrophenyl phosphate by the protein content. Results were expressed by calculating the fold changes in comparison with the control.

### 2.14. Statistical Analysis

The data were analyzed through Originpro 2023 software (Northampton, Massachusetts, USA). Data are expressed in the form of Mean ± SD. One-way (ANOVA), as well as the Bonferroni method, was used for comparison between groups. *P* < 0.05 was considered statistically significant.

## 3. Results and Discussion

### 3.1. Microstructures and Chemical Composition of Ti-Zr Alloy

**Figure 1:**
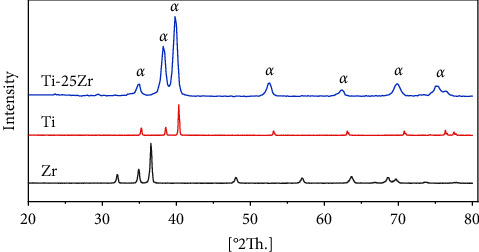
XRD patterns of pure Ti, pure Zr, and Ti–25Zr alloys.

**Figure 2:**
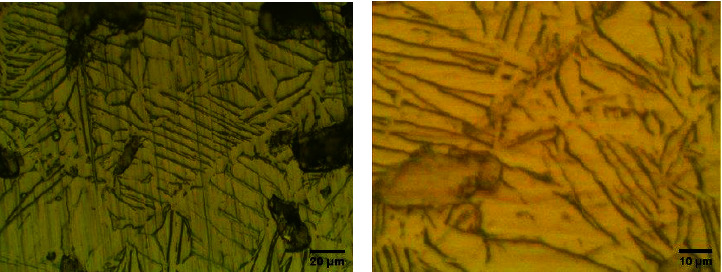
Optical microscope image of Ti–25Zr alloy surface shows *α* phase microstructure (a) at 800x, (b) at 1600x.

X-ray diffraction analysis (XRD) was used to determine the phase compositions of the alloy developed (Figure 1). shows the typical XRD profiles of Ti–25Zr alloys after 2 hours of sintering at 1300°C. The XRD data indicate that the Ti–25Zr alloy's main phase was the *α* hcp phase. The complete solid solution system of Ti and Zr may explain why Ti–25Zr alloys display the *α* phase, as shown by the optical microscope (Figure 2). In addition, the higher atomic radius of Zr (1.62 A°)

Compared to Ti (1.47 A°), the addition of Zr causes the phase lattice parameters to be raised, resulting in a shift of the peaks of the XRD chart towards a low angle. This finding agrees with those reported [31, 32].

**Figure 3:**
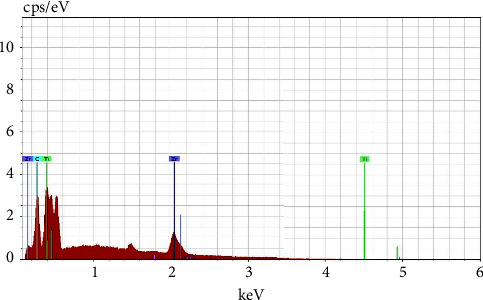
EDX spectrum for the surface of Ti-25Zr alloy.

The chemical composition and uniformity of sintered samples were evaluated using energy-dispersive X-ray spectroscopy (EDX). The findings of the semiquantitative chemical analysis conducted by the (EDX) in point are shown in (Figure 3). The EDX examination revealed the homogeneity and purity, indicating that no additional element is present in the powder combination, and (Table 1) shows the proportion of elements, revealing the appropriate mixing procedure.

### 3.2. Surface Treatment

**Figure 4:**
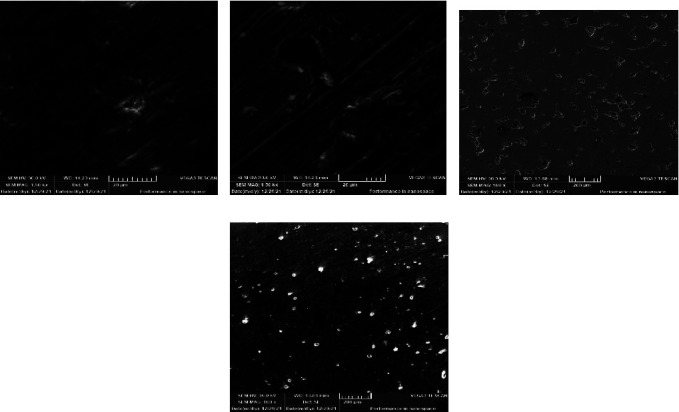
(SEM) images of the textured surface before (a, c) and after (b, d) acid and alkaline treatment.

There were apparent morphological differences between the chemically treated and untreated surfaces of the Ti-25Zr samples. Ground grooves in the surface of the control sample served as a benchmark for comparison (Figure 4(a)). The grooves were easily visible after being etched with acid and alkali (Figure 4(b)). At the same time, the pit (Figure 4(c)), after treatment, seemed to deepen and sharpen (Figure 4(d)). The chemically treated surface has more energy and is rougher, consequently enhancing the surface wettability and the coating layer adhesions.

### 3.3. Mechanicals and Physical Characterizations

Microhardness, tensile, compressive, and Modulus of Elasticity of Ti-25Zr are listed in (Table 2).

The impact of zirconium contents on the mechanical characteristics of Ti-25Zr alloys was to enhance all mechanical properties over cp-Ti, as previously indicated [31, 33, 34]. The toughness of Ti-25Zr alloys rose inversely with Zr content because the substitution of Zr resulted in crystalline lattice deformation and atomic displacement restrictions [31]. Furthermore, the Ti-25Zr alloys were complete solid solutions with hardness increases, most likely generated by solid solution hardening of the *α* phase and the contribution of the refined microstructure [35].

A modest quantity of Zr significantly improves the alloy's compressive and tensile strengths [31]. Two factors were most likely responsible for the increase in compression and tension strength caused by alloying. First, according to the Ti-25Zr alloy phase diagram, the *α* phase indicated a total solid solution with no intermetallic compound. As a result, the solid solution mechanism would create more obstacles for the slip system, increasing its mechanical properties. Second, according to the Hall-Petch formula, fine-grain strengthening increases alloy yield strength. The phase transition starting temperature decreased as Zr increased, inhibiting *α* phase expansion. Grain refining increased grain boundary area, leading to more excellent resistance to dislocation glide and improved mechanical properties [36].

### 3.4. Composite Fibers Characterization

**Figure 5:**
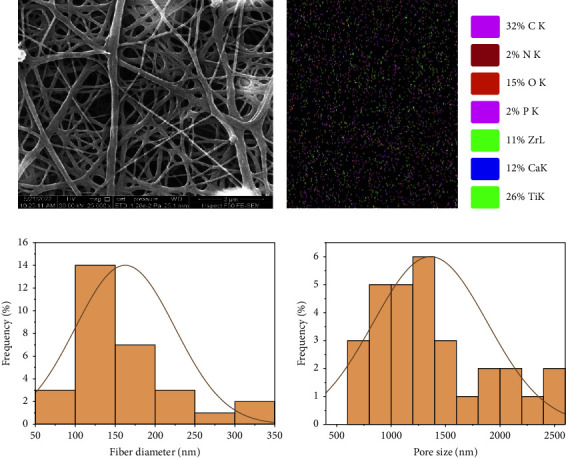
FESEM (a), EDX (b, c), fiber diameter histogram (d), pore size histogram (e) for PCL/Chitosan/Nano-CaTiO_3_ (CA1) nanocomposite fiber.

**Figure 6:**
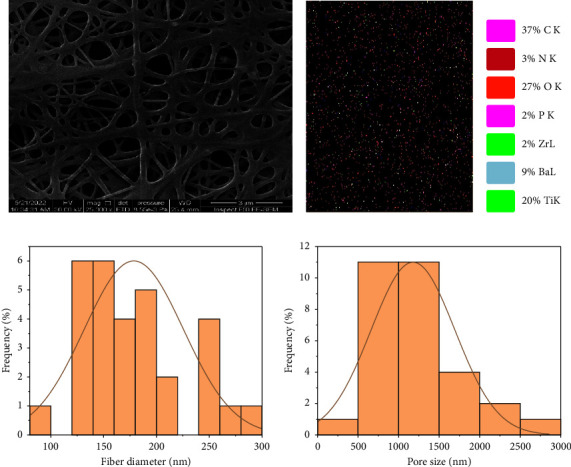
FESEM (a), EDX (b, c), fiber diameter histogram (d), and pore size histogram (e) for PCL/Chitosan/Nano-BaTiO_3_ (BA1) nanocomposite fiber.

Field-emission scanning electron microscopy (FESEM) was used to visualize the morphology of the Nanocomposite fiber. Average fiber diameter and porosity were analyzed using ImageJ, (National Institutes of Health, Bethesda, MD). Chemical elements were detected using an energy-dispersive X-ray spectrometer (EDX). Nano-fiber composite coating (CA1, BA1), bead-free homogeneously distributed nonwoven fibers can be observed (Figures 5(a) and 6(a)). The fiber diameter for (CA1) ranged from (73.88 to 334.615 nm) with a mean diameter of (162.519 ± 62.5) nm and an average size porosity of (1360.113 ± 524.52 nm) (Figures 5(d), 5(e)). EDX analysis shows that the coating layer contains calcium related to the use of (CaTiO_3_) (Figures 5(b), 5(c)).

### 3.5. Fourier Transform IR Spectroscopy

**Figure 7:**
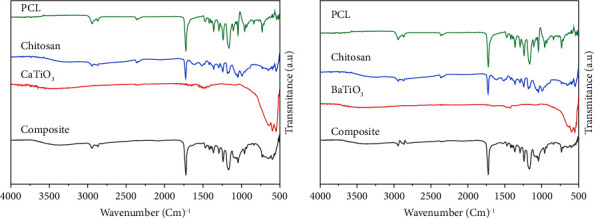
FTIR spectra of (a) pure (PCL, Chitosan, Nano-CaTiO_3_) and composite (8% w/v PCL, 2% w/v Chitosan, 1% w/v Nano-CaTiO_3_) and (b) pure (PCL, Chitosan, Nano- BaTiO_3_) and composite (8% w/v PCL, 2% w/v Chitosan, 1% w/v Nano-BaTiO_3_).

The FTIR absorption spectra of polycaprolactone, chitosan, ceramic nano-additive, and the composite coating of (PCL/Chitosan/Nano-CaTiO_3_, PCL/Chitosan/Nano-BaTiO_3_), electrospun fiber are shown in (Figures 7(a), 7(b)) respectively. The primary peak in the pristine PCL spectra is at 1723 cm^−1^, which relates to the carbonyl group of the ester group. In addition, displayed an asymmetric CH_2_ stretching peak at 2943 cm^−1^ and a symmetric CH_2_ stretching peak at 2869 cm^−1^. The absorption peak at 1294 cm^−1^ related it to the C–O and C–C stretching modes, and bands at 1239, 1161, 1107, and 1045 cm^−1^ attributed to asymmetric and symmetric C–O–C stretching [3, 25].

The intense chitosan peak was identified at 1726 cm^−1^ of carboxylate ion, and the chitosan spectra revealed a broad band of about 3245 cm^−1^ linked with O-H and N-H stretching vibrations.; peaks at 2945 and 2897 cm^−1^, corresponding to asymmetrical and symmetrical methylene groups, and an 1180–1063 cm^−1^ range, characteristic of its saccharide structure, were also detected. Interestingly, the chitosan band associated with the C=O stretching of amide I centered generally at 1615 cm^−1^. At the same time, the peak at 1510 cm^−1^ is attributed to the amideII band. In addition, the characteristic band due to the C-C aromatic stretch occurring at 1420 cm^−1^ was also present in the sample. Three peaks are situated between 1020 and 1140 cm^−1^ related to C-O-C stretching asymmetric and symmetric mode [25, 37, 38].

(Figures 7(a), 7(b)) for titanite nanocompounds (CaTiO_3_) and (BaTiO_3_), respectively, showed comparable vibration beaks with strong indication stretching band in range (410–559 cm^−1^) that attributed to the (Ti-O) [3]. A band also detected at 597 cm^−1^ corresponds to Ca-Ti-O for CaTiO_3_ and the same for BaTiO_3,_ which belongs to Ba-Ti-O [39, 40]. On the other hand, the same weak peak indicated at 1647 cm^−1^ related to the molecular water content of (O-H) band vibration shows the presence of the hydroxyl group, another weak band of hydroxylate (O-H) also present at 3378 cm^−1^ for BaTiO_3_ and at 3356 cm^−1^ for CaTiO_3_ [41, 42]. For BaTiO_3,_ the peak presented at 1430 cm^−1,^ attributed to BaTiO_3_-OH, while for CaTiO_3,_ the peak related to CaTiO_3_-OH is located at 1470 cm^−1^ [42, 43].

The FTIR for both composite coatings (PCL/Chitosan/Nano-CaTiO_3_, PCL/Chitosan/Nano-BaTiO_3_) showed a stretching vibration peak in the same range as its component with a slight shift to a low wave number, which confirms the formation of a homogenous and novel composite coat.

### 3.6. Contact Angle

The surface of a metallic implant is the primary interface between the implant and the host tissue. The adsorption of serum proteins and the adhesion behavior of osteoblasts and bacteria may be significantly affected by the hydrophilicity of the metal surface. The contact angle measurements of the coating samples and the control sample, as given in (Table 3), revealed angles ranging from 43.642° to 18.534°, indicating that the surfaces of all samples were hydrophilic.

Control sample Ti-25Zr has low wettability. However, after acid and alkaline treatment that increases surface energy and roughness, the contact angle values became significantly lower and changed from hydrophobic to hydrophilic with a contact angle of 43.642°, which is beneficial for cell adhesion and increased biocompatibility [33, 44].

The results further show that the composite coating sample's wettability improved. All the coatings were hydrophilic due to their surfaces' high porosity and roughness, the amino groups present there, and the chitosan's hydroxyl group, which is linked with the hydrogen in water molecules, which reduced their hydrophobicity [45].

As shown in (Table 3), the composite coating containing Nano(CaTiO_3_, BaTiO_3_) ceramic additive to polymer shows a low angle of 41.402° and 18.534, respectively, which may be attributed to the presence of Nano-ceramics filler particles enhance the hydrophilicity [1, 46] and, together with chitosan, improve the wettability.

### 3.7. Antibacterial Evaluation

Due to its high biodegradability, nontoxicity, and antibacterial characteristics, chitosan is often used as an antimicrobial agent, alone or in combination with other natural polymers [47, 48]. The addition of the Nano-ceramic filler (CaTiO_3_, BaTiO_3_) used with chitosan was also reported as an antibacterial material, improving the overall inhibition zone around the coating implant.

**Figure 8:**
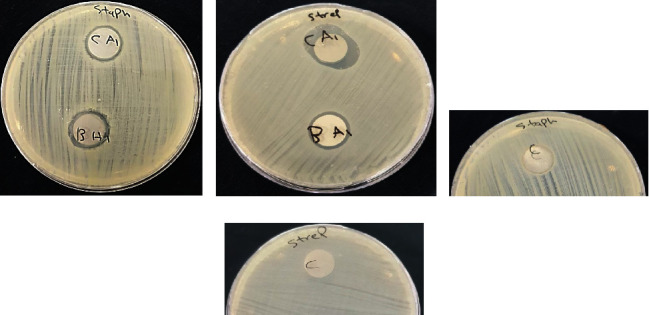
Shows the antibacterial inhibition zone of composite coating and control samples without coating in (a, c) *Staphylococcus aureus* (*S. aureus*) and (b, d) *Streptococcus mutans* (*S. mutans*).

*Staphylococcus aureus* (*S. aureus*) and *Streptococcus mutans* (*S. mutans*) bacteria were used in the antibacterial test. All the composite coatings (PCL/Chitosan/Nano-CaTiO_3_, PCL/Chitosan/Nano-BaTiO_3_) show good antibacterial effects with comparable results in both kinds of bacteria, and the results are shown in (Figure 8) and (Table 4).

Previous reports suggest using bioactive calcium titanate with incorporated silver ions, while others use ioden-containing calcium titanate to create a highly bioactive surface and simultaneously resist bacterial infection [6, 16]. Nano-BaTiO_3_ was also found to exhibit antimicrobial activity, which may be attributed to a decrease in ergosterol biosynthesis leading to cell death [49].

Furthermore, another study provides a novel approach for the electrical polarization of piezoelectric and non-piezoelectric biocompatible ceramics, including (CaTiO_3_ and BaTiO_3_), which have been investigated for the development of antimicrobial implants. On polarized surfaces, the vitality of *Staphylococcus aureus* (*S. aureus*) and *Escherichia coli* (*E. coli*) bacteria decreases dramatically. Furthermore, the effect of polarization on the antibacterial response has been investigated using a variety of mechanisms, including the formation of reactive oxygen species (ROS), catalase activity, and lipoperoxidation [50].

### 3.8. Cytocompatibility

#### 3.8.1. Cell Growth and Morphology

**Figure 9:**
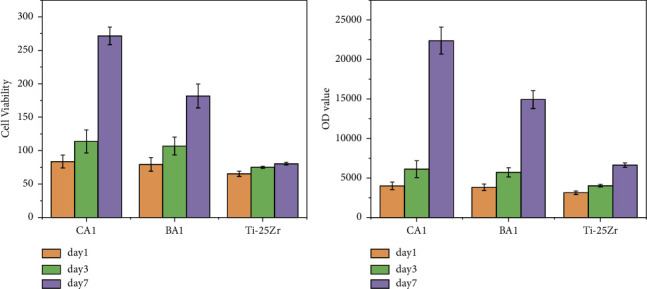
(a) The cell viability was expressed as a percentage of the number of live cells/the total number of cells (b) The proliferation activity of MC3T3-E1 cells was detected by Alamar Blue.

The cell viability, proliferation, and differentiation at the implant-host tissue interface have a significant role during implantation. Titanium and its alloys have good biocompatibility and the ability to support cell proliferation. However, because of the inert surface and the potential for microbial infection, these biometallic alloys need to activate their surfaces to promote interaction with cells and resist bacteria. The cytocompatibility of coated and noncoated Ti-25Zr alloys was studied using the MC3T3-E1 cell line and evaluated quantitatively by the Alamar Blue assay, as shown in Figures 9(a) and 9(b).

(Figure 9(a)) show the MC3T3-E1 cell viability percentage for bare Ti-25Zr alloy and the other of the same alloy with differently coated surfaces by Nano-composite fiber PCL/Chitosan/Nano-CaTiO_3_ (CA1) and PCL/Chitosan/Nano-BaTiO_3_ (BA1). As can be seen, the cell viability percentages of all the samples rise over time, rising on day 3 and reaching an even higher value on day 7. At day 7, a higher level of cell viability was observed in cells on the coated sample than in the noncoated ones (CA1 *vs.*Ti-25Zr *n* = 3, *p* < 0.0001; BA1 vs. Ti-25Zr *n* = 3, *p* <  0.00017). (CA1) coated sample displayed higher cell viability even more than (BA1) (CA1 *vs.* BA1 *n* = 3, *p* <  0.00034). This behavior may be attributable to the time taken to mineralize the coated surfaces. After 7 days of culture, the sample surface-coated (CA1) demonstrated a significantly higher value of cell viability, reaching 271.56 ± 13.15%, followed by (BA1), which has 181.63 ± 17.87%, while the noncoated bare Ti-25Zr demonstrated lower cell viability in comparison to the coated sample, with 80.52 ± 1.97%.

(Figure 9(b)) show MC3T3-E1 preosteoblast cells proliferation was assessed by determining the cell number in the scaffolds at day 1, 3, and 7, using AlamarBlue fluorescent assay. The result shows a steady increase in cellular proliferation until the higher value is reached at day 7. The (CA1) coated sample showed the highest cellular proliferation, reaching a 237.53% enhancement rate compared to the noncoated sample, followed by (BA1), which has a 125.16% enhancement rate.

This result indicates that a composite coating containing Nano-CaTiO_3_ is more conducive to cell viability and proliferation. That may be due to the fact that CaTiO_3_ can emit the Ca^2+^ ion that promotes positive reactions with cells. Calcium titanate was also reported to polarize the coated surface, enhancing cell activity [9, 50].

Incorporating BaTiO_3_ Nano-particles into a PCL/chitosan blended polymer enhanced the osteogenic differentiation of MC3T3-E1 cells. The piezoelectric effect of BaTiO_3_ induced in the Nano-composite fiber produced by the electrospinning method greatly improved the proliferation, viability, and extracellular matrix deposition of osteoblast-like cells. This observation agrees with the results reported previously, suggesting that the polymer's viscous and elastic properties play an essential role in the piezoelectric performance of piezoelectric polymer composites. With a 91.2% deacetylation degree, chitosan has piezoelectric properties and acts with barium titanite to promote cell activity [3, 23].

**Figure 10:**
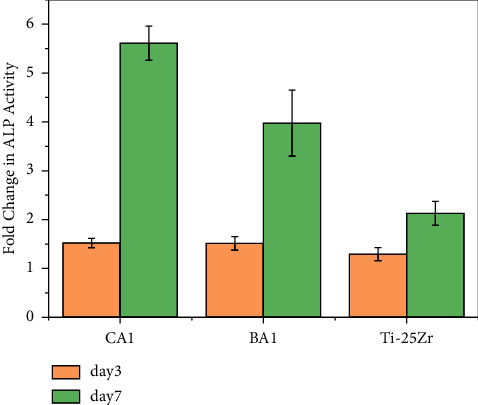
Fold change in ALP activity of MC3T3-E1 cells on different sample surfaces at 3 and 7 days of culture.

The ALP assay is a marker for time-dependent early cell differentiation. The quantitative analysis of ALP activity is shown in Figure 10. All samples' ALP activity increased with time. After 3 days, there was no significant difference in ALP activity among coated and noncoated samples, which may be attributed to the time needed for the coating layer to mineralize and react with the physiological host medium. On day 7, a higher level of ALP activity was noticed in cells on the coated sample than the noncoated one (CA1 vs. Ti-25Zr *n* = 3, *p* <  0.00022; BA1 *vs.*Ti-25Zr *n* = 3, *p* <  0.0065). (CA1) coated sample displayed higher ALP activity even more than (BA1) (CA1 vs. BA1 *n* = 3, *p* <  0.0114).

ALP is an osteogenic differentiation marker and plays a key role in reparative bone mineralization. It is expected that ALP for osteoprogenitors would be higher for surfaces with a superior biologic response. We observed an increase in ALP activity on all the surfaces over time, and (CA1) especially had the most significant influence on ALP activity on day 7. Since we observed differences in the osteoinductive properties of these surfaces, we believe that differences in surface composition and microstructure with the incorporation of a Nano-active ceramic compound in the composite coating layer play a role in the implant's osteoinductive ability, with the (CA1) alloy showing the greatest effect.

Perovskite calcium titanate CaTiO_3_ (CTO) has been used recently as the main coating component on titanium implants because of its biocompatibility, middle thermal expansion coefficient between Ti and HA, enhancement of apatite bonding, as well as its promotion of cell attachment and proliferation. CTO has demonstrated potential applications for bone regeneration due to the fact that it provides the opposite surface charges with phosphate ions in a simulated body fluid (SBF), in which it influences to grow further bone-like apatite CaTiO_3_ coatings is an effective method to enhance the biocompatibility of titanium alloy [27]. Several studies have shown that introducing calcium ions to the titanium surfaces can convert passive oxide to active oxide, resulting in a CaTiO_3_ coating and enhancing titanium's biological activity. CaTiO_3_ is also reported to induce adhesion, proliferation, and bone-like apatite deposition in osteoblasts [14, 15].

Incorporating BaTiO_3_ particles into the polymeric matrix significantly increased dielectric permittivity and decreased dielectric loss. The bioactive surface of these compounds promoted osteoblast cells' adhesion and proliferation, with distinctive ALP activity and deposition of osteocalcin and collagen I [3]. Surface composition alone can also influence cell behavior, e.g. differentiation of cells. The addition of Zr as an alloying element to Ti highlights the role of surface composition and grain refinement in the behavior of osteoblast cells, especially at the differentiation stage of cell-material interactions [33].

## 4. Conclusion

- The result shows that the Ti-25Zr alloy has a completed solid solution with *α* phase, which has better mechanical properties than pure Ti, with the exception that modules of elasticity were reduced by 29% because of the presence of Zr and porosity in the alloy. This characteristic is a favorite in biomedical applications because it reduces stress shielding.
- The electrospinning coating method produces Nanofiber with nanoroughness, high surface contact area, and porosity, which, in consequence, improves the positive reaction with the physiological medium and promotes cell adhesion, proliferation, and differentiation.
- The water contact angle shows that all samples are hydrophilic, and the contact angle is lower for the coated surface than for the uncoated one.
- The antibacterial test shows no inhibition zone for the control sample (bare Ti-25Zr) alloy, while the coated samples show a reasonable and comparable inhibition zone.
- Both (PCL/Chitosan/Nano-CaTiO_3_ and PCL/Chitosan/Nano-BaTiO_3_) coating films significantly increase cells viability, proliferation, and ALP activity of MC3T3-E1 cells on the coated surface of Ti-25Zr alloy, confirming that the electrospinning coating surface dramatically improves the cytocompatibility of the biometallic alloy.

## Figures and Tables

**Table 1:** The EDX analysis in point for the Ti-25Zr alloy.

Element	Weight (%)	Atomic (%)	Error (%)
ZrL	23.95	15.23	9.8
TiK	74.65	79.24	6.37
CK	1.4	5.53	0.38

**Table 2:** Mechanical and physical properties of Ti-25Zr alloy prepared by powder metallurgy.

Microhardness (Hv)	283 ± 21
Tension stress (MPa)	453 ± 33.4
Compression stress (MPa)	713 ± 79.3
Modulus of elasticity (GPa)	78 ± 8.1
Porosity (%)	9 ± 0.36
Apparent density	4.46 ± 0.02

**Table 3:** Contact angle image and measurements of the coated and noncoated samples.

Sample	Control Bare (Ti-25Zr)	CA1 (PCL/Chitosan/Nano-CaTiO_3_)	BA1 (PCL/Chitosan/Nano-BaTiO_3_)
Contact angle	43.542° ± 8.8	41.402° ± 9.7	18.534° ± 5.4
	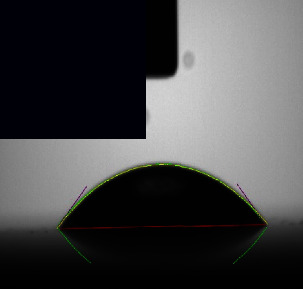	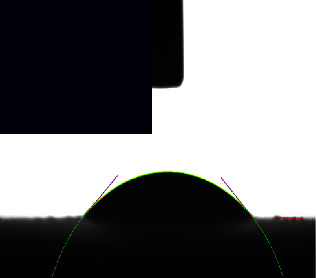	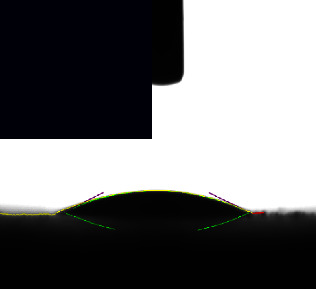

**Table 4:** Antibacterial inhibition zone measurement of the coated and noncoated samples.

	*Staph. aureus* inhibition zone (mm)	*Strep. mutans* inhibition zone (mm)
Control	0	0
CA1	17	20
BA1	18	17

## Data Availability

The data that support the findings of this study are available from the corresponding author upon request.
